# Dorsolateral Cervical Cord T2 Hyperintensity in *KIF1C* ‐Related Disease (Spastic Paraplegia 58): Two Long‐Duration Cases

**DOI:** 10.1002/acn3.70248

**Published:** 2025-11-14

**Authors:** Akihiko Mitsutake, Masao Osaki, Takashi Matsukawa, Miho Osako, Chisen Takeuchi, Hiroyuki Ishiura, Jun Mitsui, Ryo Kurokawa, Harushi Mori, Yuji Takahashi, Jun Goto, Shoji Tsuji, Tatsushi Toda

**Affiliations:** ^1^ Department of Neurology, Graduate School of Medicine The University of Tokyo Tokyo Japan; ^2^ Department of Neurology Toranomon Hospital Tokyo Japan; ^3^ Department of Neurology, Tokyo Metropolitan Kita Medical and Rehabilitation Center for the Disabled Tokyo Japan; ^4^ Department of Clinical Genetics Jikei University Hospital Tokyo Japan; ^5^ Department of Neurology Okayama University Graduate School of Medicine, Dentistry, and Pharmaceutical Sciences Okayama Japan; ^6^ Department of Precision Medicine Neurology, Graduate School of Medicine The University of Tokyo Tokyo Japan; ^7^ Department of Radiology, Graduate School of Medicine The University of Tokyo Tokyo Japan; ^8^ Department of Radiology, School of Medicine Jichi Medical University Tochigi Japan; ^9^ Department of Neurology, National Center Hospital National Center of Neurology and Psychiatry (NCNP) Tokyo Japan; ^10^ Department of Neurology International University of Health and Welfare Ichikawa Hospital Tokyo Japan; ^11^ Institute of Medical Genomics International University of Health and Welfare Chiba Japan

**Keywords:** cerebellar ataxia, hereditary spastic paraplegia, KIF1C, leukoencephalopathy

## Abstract

Pathogenic variants in *KIF1C* cause Spastic Paraplegia 58 (SPG58), typically presenting with cerebellar ataxia and spastic paraparesis. We report two unrelated patients with spastic paraparesis, cerebellar ataxia, and tremor. Whole‐exome sequence analysis identified novel homozygous variants in the motor domain of *KIF1C* (NM_006612.6): c.921G>A (p.Trp307Ter) and c.607C>T (p.Arg203Trp). In addition to the canonical brain MRI showing leukoencephalopathy with posterior dominance and hyperintensity along the corticospinal tracts, both patients showed symmetric T2 hyperintensity confined to the lateral and dorsal columns of the cervical cord. Given the long disease durations (22 and 51 years), these findings may represent late‐emerging or previously overlooked spinal cord involvement and broaden the neuroradiological spectrum of SPG58.

## 1 Introduction

Kinesins constitute a large family of microtubule‐dependent motor proteins that play a vital role in intracellular transport, which is crucial for the maintenance and function of neurons. These proteins are responsible for anterograde transport along microtubules. This process is essential for maintaining the long axons, whose integrity is critical for proper neuronal function [1]. Disruptions in these transport mechanisms cause several neurodegenerative disorders, including hereditary spastic paraplegia (HSP).

HSP comprises a clinically and genetically heterogeneous group of disorders primarily characterized by progressive spasticity and pyramidal weakness of the lower extremities. It is classified into pure and complex forms. The complex form has additional neurological features including ataxia, cognitive decline, or sensory deficits. Pathogenic variants in kinesin genes, including *KIF5A* (SPG10), *KIF1A* (SPG30), and *KIF1C* (spastic ataxia 2, also known as SPG58), have been implicated in the pathogenesis of HSP [2, 3, 4, 5, 6]. SPG58 typically presents with childhood–early‐adult onset spastic paraparesis accompanied by cerebellar ataxia; brain MRI typically shows diffuse leukoencephalopathy with symmetric T2 hyperintensity along the corticospinal tracts. Spinal involvement has been rarely reported in SPG58, with cord atrophy described in two families [5, 7]; however, dorsolateral T2 hyperintensity has not been reported.

In this report, we describe two unrelated patients presenting with spastic paraparesis and cerebellar ataxia, both harboring novel homozygous pathogenic variants in *KIF1C*. In addition to the characteristic brain MRI findings, cervical spine MRI revealed symmetric T2 hyperintensities within the lateral and dorsal columns, which may be a novel feature of SPG58.

## 2 Case Report

### 2.1 Patient 1

**FIGURE 1:**
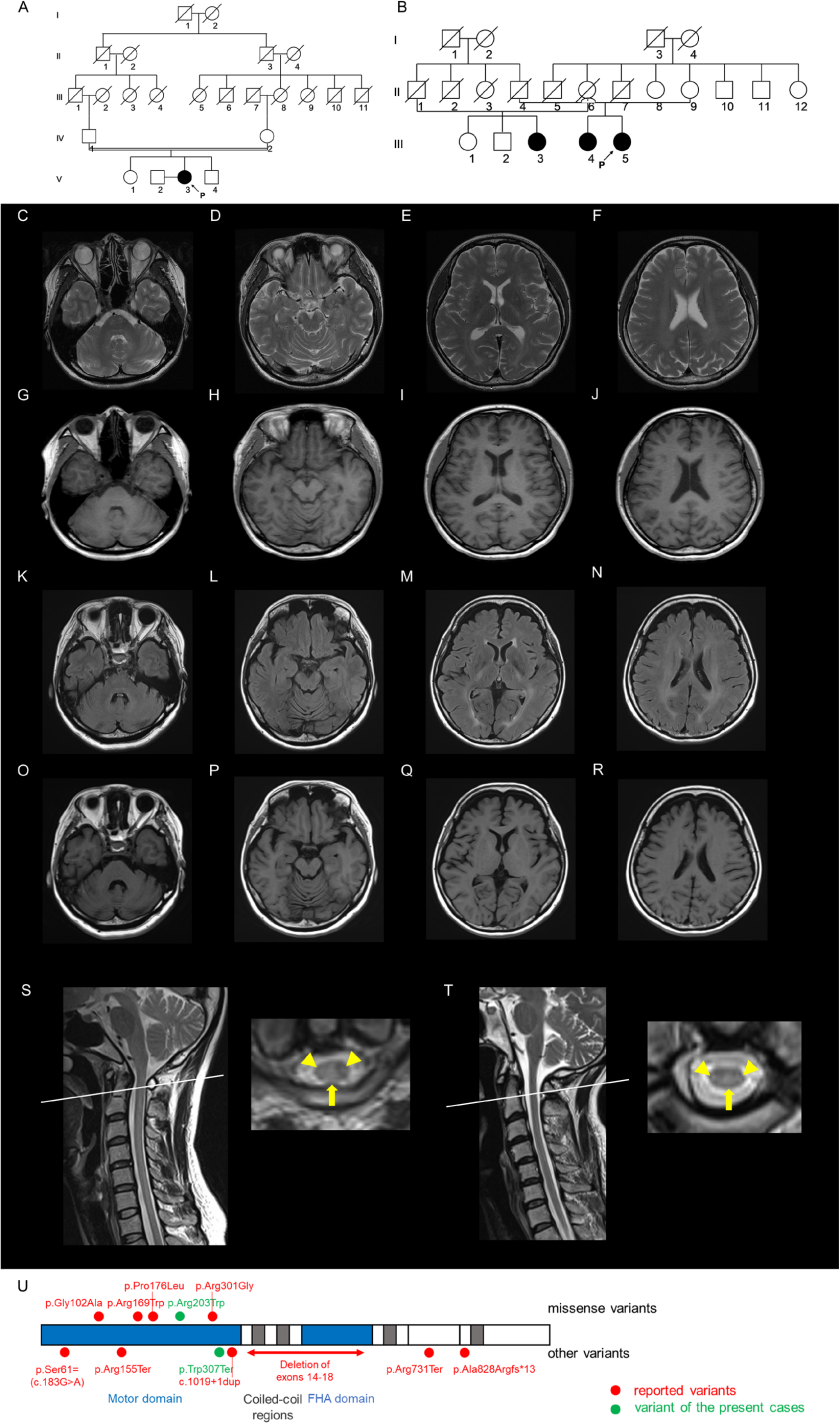
Imaging and genetic studies of Patients 1 and 2. (A, B) Pedigree charts of Patient 1 (A) and Patient 2 (B) are shown. (C–J) Brain MRI of Patient 1 at the age of 34 is shown. T2‐weighted imaging (T2WI) shows mild cerebellar atrophy, hyperintensity in the cerebral white matter with posterior dominance, and hyperintensity along the corticospinal tract (C–F). T1‐weighted imaging (T1WI) shows diffusely decreased signal intensity in the white matter, suggestive of hypomyelination (G–J). (K–R) Brain MRI of Patient 2 at the age of 55 is shown. Fluid‐attenuated inversion recovery imaging shows mild cerebellar atrophy, hyperintensity in the cerebral white matter with posterior dominance, and hyperintensity along the corticospinal tract (K–N). T1WI shows diffusely decreased signal intensity in the white matter, suggestive of hypomyelination (O–R). (S) Spine MRI of Patient 1 at the age of 34 is shown. T2WI shows hyperintensity in the lateral (arrowheads) and dorsal columns (arrow) of the cervical cord. (T) Spine MRI of Patient 2 at the age of 48 is shown. T2WI shows hyperintensity in the lateral (arrowheads) and dorsal columns (arrow) of the cervical cord. (U) Schematic representation of the KIF1C protein showing the motor, coiled‐coil, and FHA domains. Novel pathogenic variants identified in this study are indicated in green, whereas previously reported variants are shown in red.

The patient is a 34‐year‐old woman, born to her parents who are second cousins. There was no family history of neurological disorders (Figure 1A). Gait disturbance was evident in early childhood, characterized by frequent falls and difficulty in descending stairs. A postural tremor of the hands appeared when holding objects, and dysarthria was noted at age 12. By the age of 27, she began to experience spasticity in her lower extremities, and by the age of 30, incoordination became evident. As the spasticity and incoordination progressively worsened, she visited a regional hospital at the age of 33. Spastic ataxia was suspected, and she was referred to our department at the age of 34. Neurological examination revealed scanning and slurred speech, ataxia in her four limbs and trunk, spasticity in the lower extremities, bilateral Babinski signs, and postural tremor in the neck and bilateral upper extremities. Infratentorial MRI demonstrated mild cerebellar atrophy without discernible brainstem signal abnormalities. Supratentorially, T2‐weighted imaging showed hyperintensity in the cerebral white matter with posterior dominance, and along the bilateral corticospinal tract (Figure 1C–F). T1‐weighted imaging (T1WI) showed diffusely decreased signal intensity in the white matter, suggestive of hypomyelination (Figure 1G–J). Cervical spine MRI showed bilateral, symmetric lateral and dorsal column T2 hyperintensity, with relative sparing of the central gray matter (Figure 1S). Nerve conduction studies were normal.

### 2.2 Patient 2

The patient is a 55‐year‐old woman. No consanguinity in her family was evident, but the parents came from nearby rural regions. Her sibling and cousin had gait disturbance (Figure 1B). The patient began experiencing postural tremors in both hands and the head, along with an unsteady gait, around the age of four. These symptoms made activities such as jumping and running difficult. Although she consistently finished last in running races and had difficulty in jumping exercises, she graduated from high school without requiring special education support. At the age of 24, she was evaluated at a regional hospital and diagnosed with spinocerebellar degeneration. By the age of 38, she required the use of a wheelchair. At the age of 48, she was completely dependent on it, and she visited our department at the age of 50. Infratentorial MRI demonstrated mild cerebellar atrophy without discernible brainstem signal abnormalities. Supratentorially, fluid‐attenuated inversion recovery imaging showed hyperintensity in the cerebral white matter with posterior dominance, and along the bilateral corticospinal tract (Figure 1K–N). T1WI showed diffusely decreased signal intensity in the white matter, suggestive of hypomyelination (Figure 1O–R). Serial imaging showed no interval change. Spinal MRI showed T2 hyperintensity in the lateral and dorsal columns of the cervical cord (Figure 1T).

Nerve conduction studies showed mildly reduced motor and sensory nerve conduction velocities, more pronounced in the lower limbs (left median motor velocity 38.8 m/s, ulnar 40.1 m/s, tibial 36.4 m/s; median sensory velocity 42.9 m/s, ulnar 26.4 m/s, sural 27.9 m/s), with relatively preserved compound muscle action potentials (median 6.4 mV, ulnar 6.2 mV, tibial 2.5 mV) and sensory nerve action potentials (median 7.0 μV, ulnar 5.0 μV, and sural 7.0 μV), consistent with mild demyelination.

## 3 Genetic Analysis

Written informed consent was obtained from Patients 1 and 2. Genomic DNA was extracted from peripheral blood leukocytes from each patient. Whole‐exome sequence analysis was performed using the SureSelect Human All Exon V6 + UTRs kit (Agilent Technologies). Sequences were aligned with Burrows‐Wheeler Aligner (BWA), and variants were called using SAMtools [8, 9]. Rare variants were then filtered by selecting those with a quality score above 20 and a minor allele frequency below 0.01, using our in‐house exome database of 1163 Japanese controls. This resulted in the identification of 348 and 271 variants in Patient 1 and in Patient 2, respectively.

We then searched for rare variants in genes related to cerebellar ataxia (Table S1), and identified homozygous variants in *KIF1C* in these patients. These variants were located in the motor domain, a mutational hotspot [5, 6]. Patient 1 harbored c.921G>A (NM_006612.6), p.Trp307Ter (NP_006603.2). It is not listed in HGMD or ClinVar, is absent from gnomAD v4.1.0 and ToMMo 60KJPN, and shows a Combined Annotation Dependent Depletion (CADD) v1.7 Phred score of 43.0. Given that loss of function is an established mechanism in *KIF1C* [6], this variant is classified as pathogenic according to ACMG criteria (PVS1, PM1, PM2, PP4) [10]. Patient 2 harbored c.607C>T (NM_006612.6), p.Arg203Trp (NP_006603.2). The affected residue is conserved across vertebrates. The variant is not listed in HGMD or ClinVar, is absent from ToMMo 60KJPN, and is very rare in gnomAD v4.1.0 (allele frequency 4.964 × 10^−6^). It is observed only in the heterozygous state, with no homozygotes reported, which is consistent with an autosomal‐recessive disease model. CADD v1.7 Phred score was 28.4. Considering the hotspot location and the characteristic phenotype, this variant is classified as likely pathogenic (PM1, PM2, PP3, PP4) [10].

To determine the zygosity of the *KIF1C* variants, we performed copy number analysis using droplet digital PCR (ddPCR) (Figure S1A) [11]. Primer and probe sequences are provided in Table S2, and the protocol is described in Methods S1. The copy number ratio of *KIF1C* to *AP3B1* was calculated from the ratio of FAM‐positive droplets (detecting the pathogenic variant of the target gene *KIF1C*) to HEX‐positive droplets (detecting *AP3B1* as an internal control). The copy number ratios of *KIF1C* to *AP3B1* were 1.00 and 1.02 in Patients 1 and 2 respectively, confirming the homozygosity of the *KIF1C* variant in each patient (Figure S1B).

## 4 Discussion

We summarized the domain architecture of KIF1C and mapped reported variants together with the two homozygous variants identified here (p.Trp307Ter; p.Arg203Trp), both located within the motor domain (Figure 1U) [5, 6, 7, 12, 13, 14]. Pathogenic variants identified include missense, nonsense, frameshift, and splice‐site variants, as well as a large deletion encompassing exons 14 to 18. These variants predominantly localize within the motor domain of the KIF1C protein, underscoring the critical role this domain plays in its function. Consistent with previous reports, the present patients exhibited posterior‐dominant hypomyelination‐like changes on brain MRI, with corticospinal‐tract hyperintensity and mild cerebellar atrophy. Notably, both also showed symmetric T2 hyperintensity restricted to the lateral and dorsal columns of the cervical cord, a finding not previously reported in SPG58.

We next reviewed the clinical and radiological features reported in patients with KIF1C‐related disease to contextualize our findings. To ensure genetic consistency, only biallelic KIF1C cases were included in this summary. Heterozygous carriers—sometimes reported with mild symptoms—were excluded because of uncertain penetrance and expressivity. Across cohorts with available data, disease duration at last evaluation was up to 38 years, indicating a mild‐to‐moderate, slowly progressive course (Table 1). The relatively long duration in many reported cases allows assessment of potential imaging changes associated with disease progression.

**TABLE 1:** Summary of reported cases with Biallelic *KIF1C* variants and their neuroimaging findings.

Study	Case	Genotype	Onset (years)	Last eval. (years)	Brain MRI	Spinal MRI
[5]	THI26001‐3	c.305G>C; c.527C>T	18	48	WM/CST hyperintensity; cerebellar atrophy	N/A
	THI26001‐4	c.305G>C; c.527C>T	30	45	N/A	N/A
	THI26001‐5	c.305G>C; c.527C>T	18	42	WM/CST hyperintensity; cerebellar atrophy	Spinal cord atrophy
	IHG25215‐5	c.901A>G (homozygous)	10	48	N/A	N/A
[6]	V_1	c.2191C>T (homozygous)	10	24	WM/CST hyperintensity	N/A
	V_2	c.2191C>T (homozygous)	13	N/A	N/A	N/A
	V_4	c.2191C>T (homozygous)	10	12	N/A	N/A
	V_6	c.2191C>T (homozygous)	6	N/A	N/A	N/A
	II_1	c.505C>T (homozygous)	7	N/A	N/A	N/A
	II_5	c.505C>T (homozygous)	16	N/A	N/A	N/A
	II_7	c.505C>T (homozygous)	1	N/A	N/A	N/A
[7]	Family A II‐1	c.463C>T (homozygous)	3	24	WM/CST hyperintensity; brainstem/cerebellar atrophy	Cervical cord atrophy
	Family A II‐2	c.463C>T (homozygous)	4	17	WM/CST hyperintensity	Cervical cord atrophy
	Family B II‐1	c.2478delA (homozygous)	3	22	WM/CST hyperintensity	N/A
[13]	twins_1	c.1019 + 1dup (homozygous)	< 1	20	WM/CST hyperintensity	N/A
	twins_2	c.1019 + 1dup (homozygous)	< 1	20	WM/CST hyperintensity	N/A
Present study	Patient 1	c.921G>A (homozygous)	12	34	WM/CST hyperintensity; cerebellar atrophy	Lateral/dorsal cervical cord hyperintensity
	Patient 2	c.607C>T (homozygous)	4	55	WM/CST hyperintensity; cerebellar atrophy	Lateral/dorsal cervical cord hyperintensity

Abbreviations: CST, corticospinal tract; eval., evaluation; N/A, not available; WM, white matter.

Consistent with previous studies, brain MRI typically shows posterior‐dominant hypomyelination‐like changes with corticospinal‐tract hyperintensity and mild cerebellar atrophy. Notably, both patients in the present study also displayed symmetrical T2 hyperintensity confined to the lateral and dorsal columns of the cervical cord—a feature not previously described in SPG58. Among the few published cases with spinal MRI, all showed cord atrophy and represented long‐standing disease. Our observation of tract‐selective lateral and dorsal column hyperintensity, together with the canonical supratentorial pattern, suggests two non‐exclusive possibilities: (i) these cervical changes may develop with disease progression, and/or (ii) they may have been overlooked because axial cervical sequences were not routinely obtained or carefully reviewed. We propose that future studies include standardized sagittal and axial cervical MRI across disease stages to clarify how frequently this combined brain‐and‐cord pattern occurs in SPG58 and how it relates to clinical milestones.

## Author Contributions

The author takes full responsibility for this article.

## Ethics Statement

This study was approved by the institutional review board of the University of Tokyo.

## Consent

The patients provided written informed consent for publication.

## Conflicts of Interest

The authors declare no conflicts of interest.

## Supporting information


**Figure S1:** Copy number analysis of Patients 1 and 2. To determine whether the variant was homozygous or hemizygous, a copy number analysis was performed by droplet digital PCR (A). The copy number ratio was calculated as the ratio of FAM‐positive droplets (detecting the pathogenic variant of the target gene *KIF1C*) to HEX‐positive droplets (detecting *AP3B1* as an internal control). The experiment was conducted three times for biological replication (dots). The copy number ratios of *KIF1C* to *AP3B1* were calculated as 1.00 in Patient 1 and 1.02 in Patient 2. The error bars indicate ±1 standard deviation (B).
**Table S1:** A list of genes related to cerebellar ataxia.
**Table S2:** Primers and probes for digital droplet PCR.
**Methods S1**. Protocols for digital droplet PCR.

## Data Availability

The data that support the findings of this study are available on request from the corresponding author. The data are not publicly available due to privacy or ethical restrictions.
