# Supplementary material for: Dorsolateral Cervical Cord T2 Hyperintensity in KIF1C ‐Related Disease (Spastic Paraplegia 58): Two Long‐Duration Cases
Source: Ann Clin Transl Neurol. 2025 Nov 14;13(2):387–92. doi: 10.1002/acn3.70248 (PMC12883705; doi:10.1002/acn3.70248)
Supplement: Supplementary file 1 — Figure S1: Copy number analysis of Patients 1 and 2. To determine whether the variant was homozygous or hemizygous, a copy number analysis was performed by droplet digital PCR (A). The copy number ratio was calculated as the ratio of FAM‐positive droplets (detecting the pathogenic variant of the target gene KIF1C) to HEX‐positive droplets (detecting AP3B1 as an internal control). The experiment was conducted three times for biological replication (dots). The copy number ratios of KIF1C to AP3B1 were calculated as 1.00 in Patient 1 and 1.02 in Patient 2. The error bars indicate ±1 standard deviation (B). Table S1: A list of genes related to cerebellar ataxia. Table S2: Primers and probes for digital droplet PCR. Methods S1. Protocols for digital droplet PCR. [file ACN3-13-387-s001.docx]

**Supplemental Materials**

**Dorsolateral Cervical Cord T2 Hyperintensity in KIF1C‑Related Disease (Spastic Paraplegia 58): Two Long‑Duration Cases**

**Supplementary Table 1. A list of genes related to cerebellar ataxia**

*AARS2, ABCA2, ABCB7, ABCD1, ABHD12, ACO2, ADCK3, ADPRHL2, AFG3L2, AGTPBP1, AHI1, ALDH5A1, ANO10, APTX, ARL13B, ARL6, ATCAY, ATM, ATP13A2, ATP1A3, ATP2B3, ATP7B, ATP8A2, AUH, BBS1, BBS10, BBS12, BBS2, BBS4, BBS5, BBS7, BBS9, BEAN1, CA8, CACNA1A, CACNA1G, CACNA2D2, CACNB4, CAMTA1, CAPN1, CASK, CC2D2A, CCDC88C, CEP290, CEP41, CHCHD10, CLCN2, CLN5, CLN6, CLN8, CLPB, CLPP, COA7, COASY, COQ2, COQ4, COX20, CP, CPLANE1 CSTB, CTBP1, CTDP1, CTSA, CWF19L1, CYP27A1, CYP2U1, CYP7B1, DHPS, DNAJC19, DNAJC5, DNMT1, DOCK3, EBF3, EEF2, ELOVL4, ELOVL5, FA2H, FBXL4, FDXR, FGF14, FLVCR1, FMR1, FXN, GBA2, GFAP, GOSR2, GRID2, GRM1, GSS, HARS2, HEXB, HIBCH, INPP5E, IRF2BPL, ITM2B, ITPR1, KCNA1, KCNC3, KCND3, KCNJ10, KIF1C, KIF5A, KIF7, LAMA1, LARS2, LMNB1, LRPPRC, LRSAM1, MARS2, MECR, MGME1, MKKS, MKS1, MME, MRE11A, MSTO1, MTFMT, MTPAP, MTTP, NDUFAF6, NDUFS2, NDUFS4, NDUFS7, NDUFS8, NDUFV1, NKX2-1, NKX6-2, NOL3, NPC1, NPC2, NPHP1, NUBPL, OFD1, OPA1, OPHN1, PANK2, PAX6, PDYN, PEX10, PEX16, PEX2, PEX3, PEX6, PEX7, PHYH, PMM2, PNKD, PNKP, PNPLA6, POLG, PRKCG, PRRT2, PUM1, RNF216, RORA, RPGRIP1L, RUBCN, SACS, SAMD9L, SCYL1, SERAC1, SETX, SH3TC2, SIL1, SLC17A5, SLC1A3, SLC20A2, SLC25A15, SLC25A46, SLC2A1, SLC52A2, SLC9A1, SLC9A6, SNX14, SPG11, SPG20, SPG7, SPTBN2, SQSTM1, STUB1, STXBP1, SUOX, SYNE1, SYT14, TCTN1, TCTN2, TCTN3, TDP1, TDP2, TGM6, TMEM138, TMEM216, TMEM231, TMEM237, TMEM240, TMEM67, TPP1, TRAPPC11, TRIM32, TTBK2, TTC19, TTC8, TTPA, TUBB4A, TWNK, UBA5, UBTF, UCHL1, VAMP1, VLDLR, VPS13D, VWA3B, WDPCP, WDR81, WFS1, WWOX, XRCC1, ZFYVE26, ZNF423*

**Supplementary Table 2. Primers and probes for digital droplet PCR**

*KIF1C* c.921G>A, p.Trp307Ter

| Sense Primer | TCGGATTTTATCCCCTACA |
| --- | --- |
| Anti-Sense Primer | GGGGATTTGTCAGATGTATC |
| Mut Probe | GCTCACCTGACTGCTCAA |

*KIF1C* c.607C>T, p.Arg203Trp

| Sense Primer | CTCCTACGCAGACATTG |
| --- | --- |
| Anti-Sense Primer | CCCTCTGCTCCATCA |
| Mut Probe | CCTGCCTCACCATGCTTTA |

*AP3B1*

| Sense Primer | CCCATCATAATTTGTTCATTTG |
| --- | --- |
| Anti-Sense Primer | GAGCGGAATTGGAGAG |
| Mut Probe | ATACGGCACCTCGCTGACCTT |

Supplementary Methods 1. Protocols for digital droplet PCR

We prepared a 20 μL reaction mixture containing 10 μL of ddPCR™ Supermix for Probes (No dUTP) (Bio-Rad Laboratories, Hercules, CA), 1 μL of primers/probes for detecting *KIF1C* variants (primer concentration 40 μM, probe concentration 10 μM) or *AP3B1* (primer concentration 40 μM, probe concentration 10 μM), 40 ng of genomic DNA, and nuclease-free water. The prepared sample was transferred to a DG8™ Cartridge for the QX100™/QX200™ Droplet Generator (Bio-Rad Laboratories). Droplet Generation Oil for Probes (Bio-Rad Laboratories) was added, and droplets were generated using the QX200™ Droplet Generator (Bio-Rad Laboratories). The droplets were then transferred to a PCR plate, which was sealed with a foil heat seal using a PX1™ PCR Plate Sealer (Bio-Rad Laboratories) set to 180°C for 5 seconds. The sealed PCR plate was placed in an S1000™ Thermal Cycler (Bio-Rad Laboratories). The generated droplets (40 μL) were then loaded into a 96-well PCR plate (Bio-Rad) and heat-sealed with a specific aluminum foil (Bio-Rad). Thermal cycling conditions were as follows: 95 °C for 5 min, then 40 cycles of 95 °C for 30 s and 53 °C (c.921G>A) or 59 °C (c.607C>T) for 1 min (ramping rate reduced to 1.6 °C/s), and three final steps at 4 °C for 5 min, 90 °C for 5 min, and a 4 °C indefinite hold to enhance dye stabilization. After the PCR reaction, droplets were read using the QX-200 droplet reader (Bio-Rad Laboratories), and the data were analyzed using QuantaSoft Analysis Pro software (Bio-Rad Laboratories).


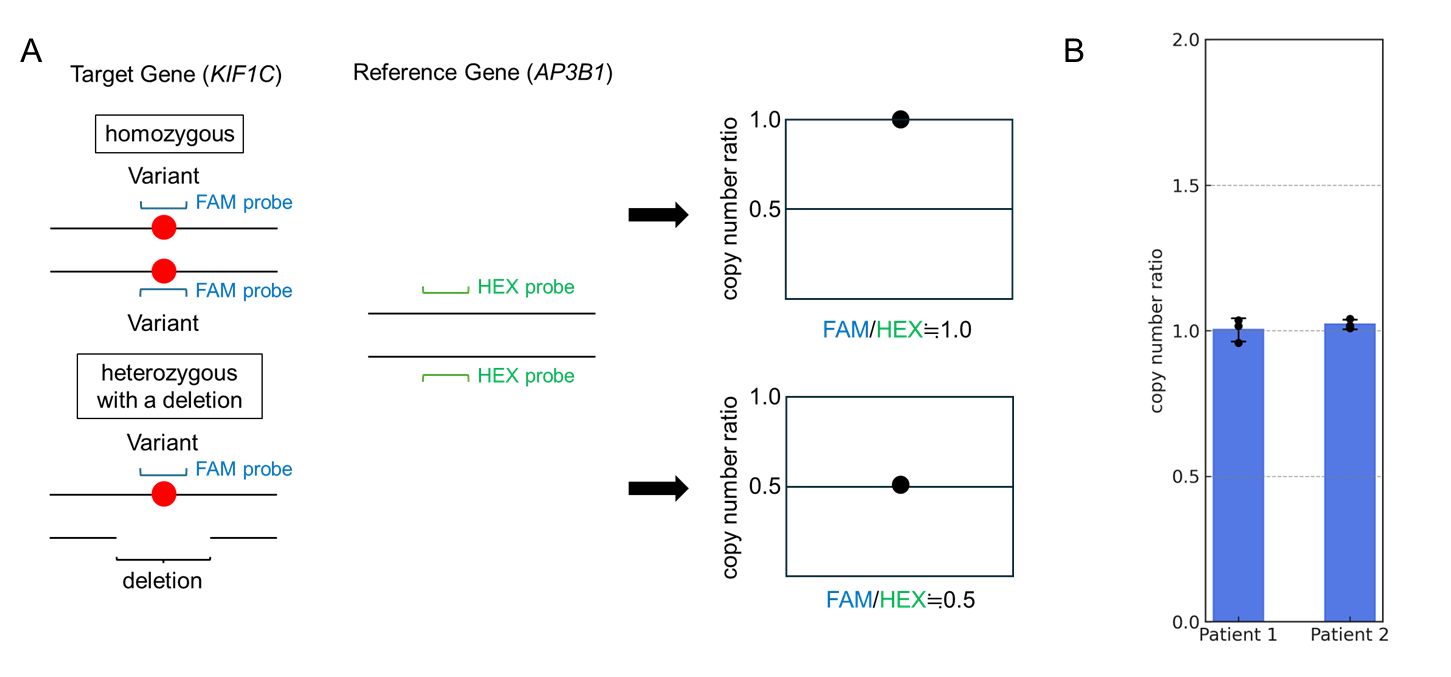


Supplementary Figure 1. Copy number analysis of Patients 1 and 2

To determine whether the variant was homozygous or hemizygous, a copy number analysis was performed by droplet digital PCR (A). The copy number ratio was calculated as the ratio of FAM-positive droplets (detecting the pathogenic variant of the target gene *KIF1C*) to HEX-positive droplets (detecting *AP3B1* as an internal control). The experiment was conducted three times for biological replication (dots). The copy number ratios of *KIF1C* to *AP3B1* were calculated as 1.00 in Patient 1 and 1.02 in Patient 2. The error bars indicate ±1 standard deviation (B).
